# Blood–brain barrier breakdown, central nervous system cell damage, and infiltrated T cells as major adverse effects in CAR-T-related deaths: a literature review

**DOI:** 10.3389/fmed.2023.1272291

**Published:** 2024-01-08

**Authors:** Fabio Del Duca, Gabriele Napoletano, Gianpietro Volonnino, Aniello Maiese, Raffaele La Russa, Marco Di Paolo, Serena De Matteis, Paola Frati, Massimiliano Bonafè, Vittorio Fineschi

**Affiliations:** ^1^Department of Anatomical, Histological, Forensic and Orthopedical Sciences, Sapienza University of Rome, Rome, Italy; ^2^Section of Legal Medicine, Department of Surgical, Medical, Molecular Pathology and Critical Care Medicine, University of Pisa, Pisa, Italy; ^3^Department of Clinical and Experimental Medicine, University of Foggia, Foggia, Italy; ^4^Immunobiology of Transplants and Advanced Cellular Therapies Unit, IRCCS Azienda Ospedaliero-Universitaria di Bologna, Bologna, Italy; ^5^Department of Medical and Surgical Sciences, University of Bologna, Bologna, Italy

**Keywords:** CAR-T, death, edema, blood–brain barrier breakdown, autopsy

## Abstract

**Background:**

CAR-T-related deaths observed worldwide are rare. The underlying pathogenetic mechanisms are the subject of study, as are the findings that enable diagnosis. A systematic literature search of the PubMed database and a critical review of the collected studies were conducted from the inception of this database until January 2023. The aim of the study is to determine when death is related to CAR-T cell therapy and to develop a shareable diagnostic algorithm.

**Methods:**

The database was searched by combining and meshing the terms (“CAR-t” OR “CART”) AND (“Pathology” OR “Histology” OR “Histological” OR “Autopsy”) AND (“Heart” OR “Cardiac” OR “Nervous System” OR “Kidney” OR “Liver”) with 34 results and also the terms: [(Lethal effect) OR (Death)] AND (CAR-T therapy) with 52 results in titles, abstracts, and keywords [all fields]. One hundred scientific articles were examined, 14 of which were additional records identified through other sources. Fifteen records were included in the review.

**Results:**

Neuronal death, neuronal edema, perivascular edema, perivascular and intraparenchymal hemorrhagic extravasation, as well as perivascular plasmatodendrosis, have been observed in cases with fatal cerebral edema. A cross-reactivity of CAR-T cells in cases of fatal encephalopathy can be hypothesized when, in addition to the increased vascular permeability, there is also a perivascular lymphocyte infiltrate, which appears to be a common factor among most authors.

**Conclusion:**

Most CAR-T-related deaths are associated with blood–brain barrier breakdown, central nervous system cell damage, and infiltrated T cells. Further autopsies and microscopic investigations would shed more light on the lethal toxicity related to CAR-T cells. A differential diagnosis of CAR-T-related death is crucial to identifying adverse events. In this article, we propose an algorithm that could facilitate the comparison of findings through a systematic approach. Despite toxicity cases, CAR-T therapy continues to stand out as the most innovative treatment within the field of oncology, and emerging strategies hold the promise of delivering safer therapies in future.

## 1 Introduction

Chimeric antigen receptor (CAR)-T cell therapy is a novel immunotherapy that has demonstrated remission responses in relapsed/refractory (R/R) hematological cancers (1, 2). CAR-T cells are derived from the patient’s own cells to overcome allogeneic rejection or graft-versus-host disease (GvHD). However, this approach requires individual, bespoke manufacturing. Autologous CAR-T cells are activated T cells transduced with a retroviral or lentiviral vector containing the CAR gene.

The first generation of CARs consisted of a transmembrane domain and an intracellular domain derived from the CD3ζ molecule of the endogenous T-cell receptor (2). Second-generation CARs contain a CD3ζ chain and an intracellular signaling domain of various costimulatory molecules, such as 4-1BB (CD137), CD28, OX40 (CD134), and induction T-cell stimulators (ICOS or CD278), which have improved the efficacy and persistence of CAR-T cells (3). Third-generation CARs have two signaling domains and a CD3ζ chain, such as CD3ζ–CD28–OX40, which, compared to second-generation CARs, provides increased activation signals, cytokine production, and anti-tumor activity in these cells (4). The latest generation of CAR-T cells is currently programmed to inhibit the tumor microenvironment (5).

CAR-T cells allow for high specificity and a sustained response that is HLA-independent, owing to the ability of these “living drugs” to bind to specific targets for which they have been programmed. The consistent expression of B-cell markers, such as CD19, CD20, and CD22 (6), or maturation antigens (e.g., BCMA) (7, 8), in many B-cell malignancies, has made them excellent targets for CAR-T cell therapy.

### 1.1 Current use of CAR-T cells

In order to address the need for new therapeutic options that ensure a high standard of safety for human health, numerous clinical CAR-T studies are being conducted worldwide (9). To illustrate, various European centers, universities, and academic institutions are actively pursuing research in this field, including studies such as NCT03373097 for neuroblastoma and NCT03373071 for CD19+ diseases, both conducted in Italy at the Bambino Gesù Pediatric Hospital in Rome. Nevertheless, the largest number of clinical trials currently takes place in the United States and China. Several clinical trials have explored CD19- and CD22-targeted CAR-T cells (NCT01044069 and NCT02315612), showing promising results in acute lymphoid leukemia (ALL), as well as CD19-targeted CAR-T cells in various non-Hodgkin lymphoma (NHL) subtypes (10–19).

Axicabtagene ciloleucel (axi-cel), INN-lisocabtagene maraleucel (liso-cell), brexucabtagene autoleucel (brexu-cel), and tisagenlecleucel (tisa-cel) represent second-generation autologous CD19-targeted CAR-T cell therapies approved for the treatment of relapsed/refractory large B-cell lymphoma (LBCL) based on phase II trials ZUMA-1 (15, 20) and JULIET (21).

More recently, several clinical trials have demonstrated promising results in patients with relapsed/refractory multiple myeloma (MM) treated with CAR-T cell therapy (8, 22–26). Various antigens, including CD33 and CD123, which are overexpressed on acute myeloid leukemia (AML) leukemic stem cells, have been extensively studied in preclinical models (27–29).

### 1.2 Toxicity of CAR-T cell infusion

In parallel with the onco-hematological success of this new therapy, the European Society for Blood and Marrow Transplantation (EBMT), the Joint Accreditation Committee of ISCT and EBMT (JACIE), and the European Hematology Association (EHA) have issued reference guidelines. These guidelines not only delineate the treatment strategies developed thus far but also encompass the most frequently encountered adverse effects associated with CAR-T cell therapy. While therapy-related toxicities are generally rare, among them, the most commonly encountered are cytokine release syndrome (CRS) and immune effector cell-associated neurotoxicity syndrome (ICANS). Typically, these toxicities manifest within the first 2 weeks (1–14 days) after infusion and may persist for days, weeks, or, in rare cases, even months. Other side effects include cytopenia, hypogammaglobulinemia, B-cell aplasia, and infection. Although death related to CAR-T therapy is uncommon, some authors have estimated a therapy-related mortality rate of 5% (2).

CRS represents the predominant complication associated with CAR-T cell treatment, occurring in 30–100% of patients. Its onset usually occurs within the first week after CAR-T cell infusion and coincides with the peak of CAR-T cell expansion. According to the grading scale established by the American Society for Transplantation and Cellular Therapy (ASTCT), grade 1 CRS entails only fever (≥38°C). Grade 2 is characterized by fever, mild hypoxia, and hypotension. Grade 3 involves fever, hypoxia requiring high-flow oxygen, and hypotension necessitating vasopressor therapy. Grade 4 presents as fever accompanied by severe hypotension, profound hypoxia requiring oxygen-positive pressure therapy (e.g., BiPAP, CPAP, and mechanical intubation), corticosteroid drugs, and tocilizumab (30). Due to gravity, treatment for CRS (cytokine release syndrome) is initiated when the grading reaches ≥G2.

ICANS is a neurotoxic syndrome occurring in 67% of patients with leukemia and 62% of patients with lymphoma treated with CD19-targeted CAR-T cell therapy. It is characterized by symptoms such as aphasia, tremor, dysgraphia, and lethargy, which can progress to global aphasia, seizures, obtundation, stupor, and coma (30). The risk of developing high-grade ICANS can be attributed to factors such as the pre-treatment tumor immune microenvironment, elevated levels of inflammatory cytokines (e.g., IL-6), C-reactive protein (CRP), ferritin, and D-dimer, as well as the infused product (e.g., CAR-T cells with CD28 as a costimulatory domain) and pre-existing central nervous system (CNS) disorders (31). It has been reported that high fever and neurological symptoms in the first few days after infusion can predict severe ICANS (6). ICANS gravity is established by a score system (CARTOX-10), which allows for the classification of severity into four grades (0–3).

Understanding the pathophysiological mechanisms underlying ICANS is of significant importance and will help interpret the histopathological findings to date. It appears that the symptomatic manifestation is primarily driven by increased vascular permeability linked to elevated cytokines associated with endothelial cell activation. Additionally, macrophage activation syndrome (MAS) and tumor lysis syndrome (TLS) have been documented as side effects of therapy. In this highly pre-treated population, prior cardiotoxic chemotherapy or underlying cardiac conditions in these patients warrant assessment. By the way, some authors have reported cardiovascular complications in approximately 20% of CAR-T therapy cases, including hypotension, arrhythmias, myocardial damage, and even death from heart failure.

In the literature, adverse effects related to CAR-T cell therapy (ICANS, CRS, TLS, and infections) have been reported even several months after treatment. Some authors suggest that the potential risk of developing cardiovascular toxicity should prompt more stringent surveillance protocols (32). Given the growing utilization of these drugs in oncology, it is imperative for pathologists to possess knowledge of the forensic implications associated with CAR-T therapy, particularly in cases involving medical negligence or issues related to the administration of CAR-T therapy.

The objective of this study is to gain insights, through a systematic review of the scientific literature, into the elements that define CAR-T-related deaths and to highlight findings from cases involving autopsies and/or histopathological investigations.

## 2 Materials and methods

**Figure 1:**
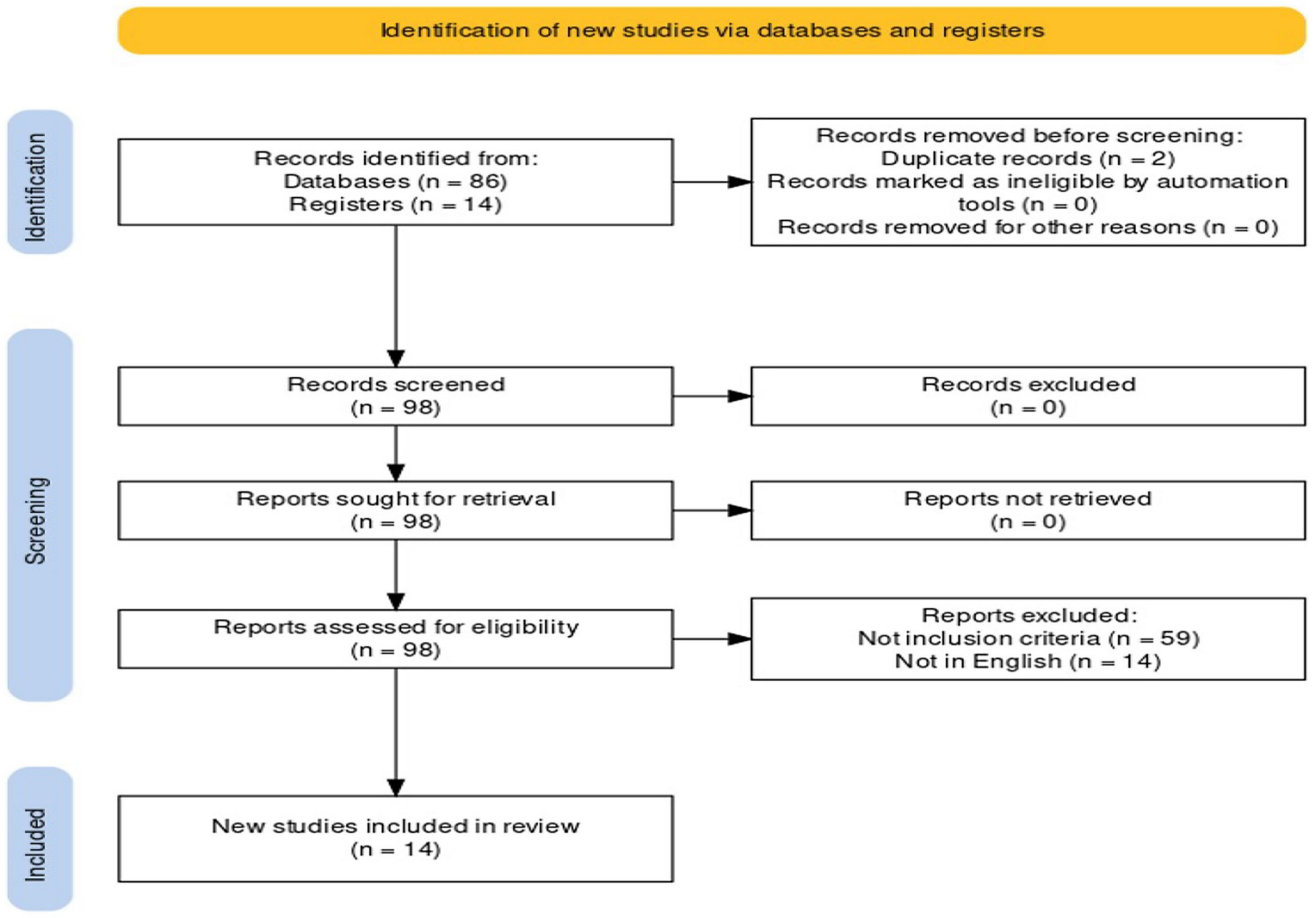
Preferred reporting items for systematic review (PRISMA) flow chart—search strategy. Study designs comprised retrospective and prospective studies, original articles, and reviews. An appraisal based on titles and abstracts as well as a hand search of reference lists were carried out. A total of 15 studies fulfilled the inclusion criteria.

This systematic review was conducted from the inception of these databases until January 2023 and followed the Preferred Reporting Items for Systematic Reviews and Meta-Analyses (PRISMA) guidelines (33). A systematic literature search of the PubMed database and a critical review of the collected studies were conducted. The database was searched by combining and meshing the terms: (“CAR-T” OR “CART”) AND (“pathology” OR “Histology” OR “histological” OR “autopsy”) AND (“heart” OR “cardiac” OR “Nervous System” OR “kidney” OR “liver”) with 34 results. We also searched the terms: [(lethal effect) OR (death)] AND (CAR-T therapy) with 52 results in titles, abstracts, and keywords [all fields]. The references of all identified articles were examined and cross-referenced to further isolate the relevant literature. A methodologic appraisal of each study was conducted, including an evaluation of bias. The data collection process included study selection and data extraction. This study was exempt from institutional review board approval as it did not involve human subjects. A waiver of patient consent was provided due to the use of deidentified patient data. Inclusion criteria included case reports, research articles, autopsy series reports, and papers describing CAR-T-related deaths. Exclusion criteria included items that did not have deaths related to CAR-T therapy; deaths related to underlying disease or leukocyte depletion/aplasia infections were excluded (Figure 1).

### 2.1 Strengths and limitations

This systematic review possesses several strengths, one of which is the international scope of the scientific literature it encompasses. In fact, the studies investigating CAR-related toxicities and deaths originate from various countries across different regions of the world. It must be noted that this review includes studies that have few cases. Therefore, the information that emerges must necessarily be updated by increasing the number of cases. There is also a gap between the number of clinically described CAR-T cell-related deaths and the number of autopsies and histopathological investigations performed.

## 3 Results

The literature research has uncovered significant involvement of the central nervous system, with a few cases of cardiotoxicity. In the investigation, the primary causes of death were neurotoxic, followed by cardiotoxicity, as reported in Table 1. As illustrated below, cerebral involvement was present in most autopsy cases.

**Table 1:** Summary of data obtained from the 14 found articles.

References	Type of tumor	Target	CAR-T**-**related death	Neurotoxicity (no. of cases)	Cardiotoxicity	Other causes of death
Gust et al. (34)	N/A	CD19	Yes	4	0	0
Neelapu et al. (20)	LBCL	CD19	Yes	0	1	1
Abramson et al. (17)	LBCL	CD19	Yes	0	0	7
Benjamin et al. (35)	ALL	CD19	Yes	0	0	1
Afzal et al. (36)	Endometrial carcinosarcoma	Not reported	Yes	1	1	0
Torre et al. (37)	N/A	CD19	Yes	N/A	N/A	N/A
Nie et al. (38)	MCL	CD19	Yes	1	1	0
Berdeja et al. (39)	MM	BCMA	Yes	1	0	5
Jung et al. (40)	LBCL	CD19	Yes	0	0	0
Karschnia et al. (41)	N/A	CD19	Yes	1	0	0
Goldman et al. (42)	N/A	CD19	Yes	0	1	1
Jacobson et al. (43)	NHL	CD19	Yes	0	0	1
Pemmaraju et al. (44)	BDCN	CD123	Yes	1	1	0
Torre et al. (45)	N/A	CD19	Yes	1	1	0
				10	6	16

Data show that CAR-T with CD-19 targeting has been associated with a higher number of deaths (18, 21, 35, 39, 43, 44, 46). This is probably due to its more extensive use compared to other, more recent drugs. Out of a total of 32 observed deaths, 22 were associated with CAR-T infusion targeting CD-19. Among these, 7 were associated with neurotoxicity, 4 with cardiotoxicity, and 11 with other causes. Schematically, Tables 2, 3 reported the elements that allowed the individual authors to correlate the deaths of the subjects examined with the infusion of CAR-T cells. In 6 out of 14 studies, patients experienced ICANS (immune effector cell-associated neurotoxicity syndrome), while three studies reported CRS (cytokine release syndrome). When brain MRI examinations were documented, they revealed brain swelling, vasogenic or cytotoxic edema, blood–brain barrier damage, and multifocal hemorrhages. For those cases where cerebrospinal fluid examinations were reported, elevated cytokine levels were observed in the presence of CD4, CD8, and CAR-T T cells (as shown in Table 3).

**Table 2:** Clinical presentation and instrumental analysis performed by the authors.

References	Death after CAR-T infusion (mean time)	Clinical symptoms	Instrumental analysis
Gust et al. (34)	3 days to 4 months after infusion	ICANS and multiorgan failure	RMI: fatal neurotoxicity; T2/FLAIR changes indicative of vasogenic edema, leptomeningeal enhancement, and/or multifocal microhemorrhages. Breakdown of BBB
Neelapu et al. (20)	Not reported	Not reported	Not reported
Abramson et al. (17)	Not reported	Not reported	Not reported
Benjamin et al. (35)	Not reported	Not reported	Not reported
Afzal et al. (36)	12 h after infusion	High-grade CRS, cardiotoxicity, hypotensive tachycardia and hypoxia	Not reported
Torre et al. (37)	<7 days from infusion	ICANS	CT: diffuse cerebral edema
Nie et al. (38)	20 days after infusion for cardiac arrest during hemodialysis.	ICANS	CT and RMI revealed cerebral edema (days 4–8). MRI showed restriction abnormalities consistent with cytotoxic swelling or BBB leakage
Marker et al. (46)	8 months after the first infusion and 2 months after the second.	ICANS/CRS	RMI 1 month prior to death demonstrated diffuse failure of CSF signal suppression on the T2 FLAIR sequences, which was concerning for an inflammatory or infectious process. In addition, showed symmetric edema within the brainstem, medial cerebellar hemispheres, and portions of the deep gray nuclei
Berdeja et al. (39)	Not reported	Not reported	
Jung et al. (40)	9 months	ICANS	Autoimmune encephalitis in the brain stem and cerebellum
Karschnia et al. (41)	53 days	ICANS	MRI: symmetrical white matter lesions extending along the posterior horn of the lateral ventricles to the basilar part of the pons. Sever pontine swelling with diffusion restriction, indicating profound edema of the brain stem due to blood–brain barrier disruption
Goldman et al. (42)	Not reported	Not reported	Not reported
Jacobson et al. (43)	Not reported	Not reported	Not reported
Pemmaraju et al. (44)	9 days after infusion	Severe clinical CRS/respiratory failure	TC: pleural effusion and lung inflammation
Torre et al. (45)	Not reported	Not reported	Not reported

LBLC, large B-cell lymphoma; MM, multiple myeloma; BCMA, B-cell maturation antigen; BDCN, blastic plasmacytoid dendritic cell neoplasm; MCL, mantle cell lymphoma.

### 3.1 Autopsy findings and histological examination

Despite the little information deduced from the scientific data examined, the review of the literature of autopsy cases of CAR-T-related death allows to know the consequences of adverse events related to therapy. The damages described by the MRI were also observed in autopsies.

#### 3.1.1 CNS verbal description and main features

The primary information gleaned from autopsy reports concerns the involvement of the central nervous system (CNS) in CAR-T-related deaths, characterized by an enlargement of cerebral volume with augmented convolutions observed *in situ*. Following dissection, the key features that could be observed include small petechial hemorrhagic areas, especially in caudal regions, the cerebellar zone, and intraparenchymal spaces such as the medulla, pons, midbrain, posterior thalamus, and cerebellum. These microscopic findings confirmed signs of CAR-T-induced encephalopathy and were observed in the absence of any herniation.

Cerebral infarcted areas, characterized by large necrotic and plaque-like lesions, are frequently described. These gross features were confirmed during the histological examination. As seen in MRI scans, acute cerebral edema was present in all cases of neurotoxicity. Notably, edema constitutes the primary histological finding, manifested as optically empty vacuoles in which cells are immersed, along with dilatation of perivascular spaces (vasogenic and cytotoxic edema).

Microscopic examinations revealed multifocal microhemorrhages and patchy parenchymal necrosis. In association with small areas of infarction, severe vascular lesions were observed, featuring karyorrhexis, fibrinoid vessel wall necrosis, and perivascular CD8+ infiltration. In one case, the authors highlighted a significant activation of macrophages/microglia (CD68+). Most slides exhibited the presence of lymphocytes, CAR-T cells, macrophages, and signs of endothelial damage.

#### 3.1.2 Cardiac involvement related to CAR-T therapy

Examination of the heart revealed hypertrophic changes with volume enlargement in both ventricles. In one case, coronary atherosclerosis was observed, but it was not linked to CAR-T therapy-related death. Histological examination showed monocyte infiltrates and lipofuscin pigmentation around some nuclei, associated with interstitial edema in the right ventricle. Scattered mast cells CD117+ were also observed throughout the myocardium, along with patchy lymphocytic infiltrate CD3+ (no CAR-T), focal monocyte necrosis, and perivascular fibrosis. Cardiac findings do not always allow for a diagnosis of cardiac death.

## 4 Discussion

CAR-T cell therapy has demonstrated a significant and long-lasting response in different types of tumors. However, if, on the one hand, CAR-T cells meet cancer cells, inducing their apoptosis, on the other, the release of inflammatory cytokines and the recruitment of myeloid cells lead to systemic inflammation that, in some cases, induces damage to healthy tissues, causing the death of the subject. The high presence of circulating inflammatory cytokines and endothelial activation driven by myeloid cells can determine the dysfunction of the blood–brain barrier (BBB) and ICANS. Capillary leak syndrome, hypotension, organ failure, disseminated intravascular coagulation, and macrophage activation syndrome have been observed in treated patients developing ICANS. Aberrant macrophages were found around the brain vasculature in a case study of fatal CAR-T-associated ICANS (45). These mechanisms would agree with what some authors have found: high levels of glial fibrillary acidic protein (GFAP), S100 calcium-binding protein B, white blood cells, protein, interferon-gamma, granzyme, granulocyte–macrophage colony-stimulating factor, macrophage inflammatory protein-1α, and TNF-α in the cerebrospinal fluid of patients experiencing ICANS (34, 45).

### 4.1 Post-mortem examination of fatal neurotoxicity

In patients with severe ICANS, an evolution to fatal cerebral edema has been observed, characterized by swollen brain with flattened gyri, ventricles, cerebral aqueduct, and narrowed sulci; however, no herniations or hemorrhages of Duret have been observed. Both at the level of the cortex and the white matter of the brain, brainstem, and cerebellum, some areas of reddish-brown color have been highlighted and confirmed as intraparenchymal hemorrhagic extravasations (31, 41, 46), sometimes in the context of frank multifocal necrotizing leukoencephalopathy (46).

From a histopathological and immunohistochemical point of view, the findings highlighted in ICANS cases are neuronal death, neuronal edema, perivascular edema, perivascular and intraparenchymal hemorrhagic extravasation, as well as perivascular clasmatodendrosis in cases of fatal cerebral edema (37, 45); at H&E staining, dilations of perivascular spaces present infiltration of inflammatory cells surrounded by eosinophilic amorphous matrix, indicative of fluid extravasation (31, 41). In some cases, areas characterized by the presence of ACL-, CD3-, and CD68-positive inflammatory cells have also been observed (40). Immunohistochemistry stains for GLUT1 were used to highlight endothelial damage, while staining for GFAP showed damage to the astrocyte component in both white matter and the cortex.

According to Gust et al. (31), severe neurotoxicity related to CAR-T cells is also characterized by intraparenchymal hemorrhages, fibrinoid vessel wall necrosis, intravascular microthrombi, infarctions, and infiltrations by CAR-T cells. These last findings find confirmation in animal models (microthrombi, microinfarcts, and infiltrations of intraparenchymal and intrameningeal CAR-T cells). In the literature, there are also cases of inflammation extended to the trunk, limbic system, and cerebellum, characterized by astrocyte damage, macrophage/microglia activation, and diffuse and scattered infiltrates of cytotoxic T lymphocytes (e.g., CD8+ at hippocampal level), where the extraction of CAR-T cell DNA from the most affected areas has allowed a causal correlation (CAR-T—brain damage, concluding for CAR-T-cell-triggered encephalitis) (40). A more extensive encephalic involvement would also be confirmed by other authors, where vacuolization and axonal spheroids in the pontine white matter, a finding consistent with Wallerian degeneration, have also been documented through LFB-PAS stains (41).

In some cases, the areas of encephalopathy were considered with caution since they could not be uniquely related to the infusion of CAR-T; these findings are also present in cases of encephalopathy or multifocal leukoencephalopathy linked to chemotherapy, hypoxia, AIDS, JC viruses, drugs, genetic factors, etc., which were excluded before correlating deaths to CAR-T cell infusion.

**Figure 2:**
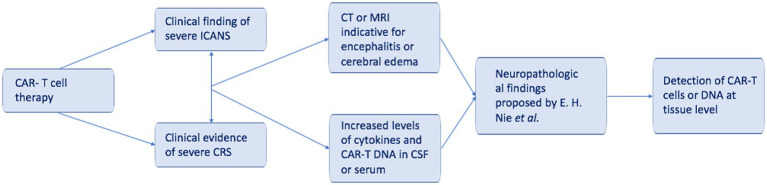
A simplified algorithm for CAR-T-related death.

The authors who have dealt with the topic so far agree with the hypothesis of cross-reactivity of CAR-T cells in cases of fatal encephalopathy when, in addition to the increase in vascular permeability, there is also a perivascular lymphocyte infiltrate (31, 37, 40, 41, 47, 48). A recent study (January 2023) proposes a multi-phase model that would explain a common mechanism underlying neuropathological findings and the greater severity of ICANS in cases of oligodendrocytic cell line involvement (38). The latter provides a clear illustrative representation of the mechanisms underlying CAR-T-related deaths, making it a useful guide in forensic cases. An algorithm proposal for the diagnosis of CAR-T-related deaths is shown in Figure 2.

**Table 3:** Collection data of autopsy and microscopic evaluation according to the liquor and plasma concentration of protein and inflammatory cells.

References	Liquor (CSF)/plasma	Autopsy	Histopathology/immunohistochemistry
Gust et al. (34)	CSF: CD4+, CD8+ CAR-T cells, and protein; during the acute neurotoxicity the concentrations of IFN-γ, TNFα, IL-6, and TNFR p55 increased.	One died from a multifocal brainstem hemorrhage and edema with DIC; 2 from acute cerebral edema, and one died from cortical laminar necrosis. In 2 cases, examinations showed multifocal microhemorrhages and patchy parenchymal necrosis in the pons, medulla, and spinal cord. Associated with small areas of infarction were more severe vascular lesions with karyorrhexis, fibrinoid vessel wall necrosis, and perivascular CD8+ infiltration.	Most of the T cells in the brain were CAR-T cells, in the pons expressing the EGFR transduction marker (51.9% of CD4−/CD8+ CAR-T cells; 48.1% of CD4+/CD8-CAR-T cells)
Neelapu et al. (20)	CSF sampling revealed elevated protein (933 mg/dL) and only a single white blood cell	Petechial hemorrhages over the bilateral cerebellar tonsils, bilateral anterior–superior cerebellar hemispheres, bilateral cerebral peduncles, optic chiasm, optic tracts, and mamillary bodies. No herniations. On section of the fresh brain, additional petechial hemorrhages were found on the superficial aspects of the medulla, pons, midbrain, posterior thalamus, and cerebellar peduncles. The lesion was hypocellular without significant inflammatory and with frequent axonal spheroids.	Rare T cells were present on CD20 and CD3 immunostaining. Neurofilament and amyloid precursor protein stains showed partially preserved neurofilament but severe axonal damage within the vacuolated lesions. CD31 stain showed decreased endothelial cells. TUNEL staining was positive in endothelial cells and possible residual microglia within the lesion.
Abramson et al. (17)	Not reported	Not reported	Not reported
Benjamin et al. (35)	Not reported	Not reported	Not reported
Afzal et al. (36)	Not reported	Heart revealed hypertrophic changes in monocytes of both ventricles along with lipofuscin pigmentation around some nuclei. Interstitial edema in right ventricle; scattered mast cells throughout the myocardium, patchy lymphocytic infiltrate along with focal monocyte necrosis and perivascular fibrosis. Lung sections showed multiple foci of platelet thrombi.	Cluster of differentiation CD3 cells was observed scattered within the myocardium in sections from the right ventricle but not T cells. CD117 was used for mast cells.
Torre et al. (37)	CSF obtained at the time of autopsy was pluricellular without a clearly defined lymphoid.	Autopsy showed severe edema, and H&E showed dilatation of the perivascular spaces with the presence of an acellular eosinophilic substance that stained for factor VIIIA and fibrin, indicating perivascular fluid extravasation. No histological findings of infections.	Staining for GLUT1 showed aberrant stain in the endothelial cells in scattered vessels, suggesting endothelial damage. GFAP showed astrocyte injury. No CAR-T cells were detected in CNS tissue.
Nie et al. (38)	CSF showed atypical lymphocytes with large nucleus, lymphocytic histiocytosis, and CAR-T lymphocytes. Lymphocytes were almost exclusively CD19 CAR-T cells with >50% of the T cells labeling CD4 + .	The external brain examination showed severe edema and multiple well-demarcated plaque-like lesions found in the centrum semiovale on coronal sections. At H&E stain lesions were limited to cerebral white matter and were composed of a dense collection of foamy macrophages with frequent axonal spheroids and severe vacuolization of the involved parenchyma.	Revealed multifocal demyelinating leukoencephalopathy. Luxol fast blue special stain showing loss of myelin in multiple well-circumscribed lesions magnification. CD163 immunostaining highlights macrophages within the demyelinating lesions. Sv40 ruled out the JC virus. There was no evidence of infection. Oligo2 and PDGFRα stain showed loss of oligodendrocyte cells.
Marker et al. (46)	CSF collected at autopsy showed high levels of CAR-T cells.	Petechial hemorrhages over the bilateral cerebellar tonsils, bilateral anterior–superior cerebellar hemispheres, bilateral cerebral peduncles, optic chiasm, optic tracts, and mamillary bodies. No herniations. On section of the fresh brain, additional petechial hemorrhages were found on the superficial aspects of the medulla, pons, midbrain, posterior thalamus, and cerebellar peduncles. The lesion was hypocellular without significant inflammatory and with frequent axonal spheroids.	Rare T cells were present on CD20 and CD3 immunostaining. Neurofilament and amyloid precursor protein stains showed partially preserved neurofilament but severe axonal damage within the vacuolated lesions. CD31 stain showed decreased endothelial cells. TUNEL staining was positive in endothelial cells and possible residual microglia within the lesion.
Berdeja et al. (39)	Not reported.	Not reported	Not reported
Jung et al. (40)	CSF: IL-1, IL-6, CAR-T cell DNA;	CAR-T cell brain infiltration (brain stem, cerebellum, and limbic system).	Severe loss of pyramidal cells in the hippocampus. In several affected areas, prominent glial-fibrillar astrocytosis is associated with massive macrophage/microglia (CD68+) activation and diffusely dispersed infiltrates of cytotoxic T lymphocytes. Negative SV-40 stain ruled out multifocal leukoencephalopathy. CAR-T cell DNA in the most affected areas suggests that patient died of severe CAR-T-cell-triggered autoimmune encephalitis.
Karschnia et al. (41)	CSF: Elevated protein levels of 90 mg/dL	Edema and increased total brain weight. Occipital and infratentorial swelling with multiple intraparenchymal hemorrhages (>pons).	In pontine tissue, enlarged perivascular spaces with fluid extravasation are consistent with blood–brain barrier dysfunction. Accumulation of intramural and perivascular CD3+/CD8+, CD3+/CD4−/CD8−, and CAR-T cells in pontine tissue; In pontine white matter axonal injury with vacuolization and spheroid formation (LFB-PAS stain) was also displayed and accompanied by microglia activation (stain CR3/43).
Goldman et al. (42)	Not reported	Not reported	Not reported
Jacobson et al. (43)	Not reported	Not reported	Not reported
Pemmaraju et al. (44)	Plasma: IL6, IL-8, IL10, IFN-γ, CCL3, CCL4	Myocardial hypertrophy, coronary atherosclerosis, cirrhotic liver, incidental prostate adenocarcinoma, and thyroid adenoma. No definitive cause of death	Decreased lymphocytes in the spleen and lymph node. Immunohistochemical stains show that the majority of lymphocytes in the lymph node are CD4+ T cells; endothelial cells in the lymph node, myocardium, and spleen are positive for CD123; in lung macrophages are positive for CD123.

Emphasizing the significance of correlating clinical data with both pre- and post-mortem instrumental analyses in conjunction with autopsy findings is crucial. For an exhaustive examination of the central nervous system, the authors suggest the simultaneous performance of cerebral evisceration and *vertebral specum* dissection. Subsequently, these specimens should be assessed as a unified entity following formalin fixation, and then examined microscopically.

### 4.2 Role of post-mortem neuroimaging—recent updates

In addition to histopathology, magnetic resonance imaging (MRI) is applied in the post-mortem examination of CAR-T-related neurotoxicity. Further studies are needed to estimate the correlation between CAR-T and brain toxicity, as it is a matter of fact that MRI could see the common findings of neurotoxicity (49, 50). A recent review underlines the role of MRI in stressing on how the infusion of CAR-T may result in an impairment of the blood–brain barrier, with signs of demyelination, micro-thrombosis, microhemorrhages/infarcts, edema, and expansive lesions related to infiltrations of intraparenchymal and intrameningeal CAR-T cells (51). It is of the utmost importance that future experimental studies give a thorough description of the post-mortem features in the neuroimaging of ICANS.

### 4.3 Epicrisis

The data collected during forensic investigations with these points can help the neuropathologist to have sufficient evidence to support a CAR-T-related death. Although the toxicity of CAR-T on the cardiovascular system is known, supporting a causal correlation appears more difficult when autopsy and immunohistochemistry do not reveal relevant elements (52, 53).

## 5 Conclusion

To diagnose CAR-T therapy-related deaths, it is crucial to have access to the patients’ clinical history and knowledge of the timing of CAR-T cell treatment. This is especially important because, to date, the number of reported deaths in this context is limited, and microscopic evidence is not pathognomonic. Distinguishing the effects of the chemotherapeutics used and opportunistic viruses from those linked to CAR-T therapy is of paramount importance. Most CAR-T therapy-related deaths manifest with blood–brain barrier disruption and damage to the central nervous system cell components, along with T-cell infiltration, as per the observed mechanism.

This study presents highly significant advancements as it enables clinicians to provide valuable information for distinguishing post-mortem deaths resulting from the therapy from those due to the natural course of the underlying disease. In this context, the remnants described can provide valuable insights into understanding the life-threatening consequences of the clinical situation and detecting them early. These findings can be used by the scientific community to investigate new methods for diagnosing fatalities associated with CAR-T therapy.

However, conducting additional autopsies and microscopic investigations would provide further insights into this subject. The investigation of CAR-T DNA presence in directly affected tissues presents an intriguing avenue for research that should be rigorously explored, validated, and potentially standardized in future (54–57). In the field of forensics, a more comprehensive understanding of CAR-T therapy-related deaths would help in determining the causes of death and resolving issues related to any legal disputes. From a forensic perspective, another intriguing area to explore is the possibility of detecting CAR-T cells even several years after treatment. This information could prove valuable and guide investigations in cases of personal identification.

## Author contributions

FD: Formal analysis, Writing – original draft. GN: Writing – original draft. GV: Writing – original draft. AM: Conceptualization, Project administration, Writing – original draft. RR: Conceptualization, Investigation, Writing – original draft. MP: Data curation, Writing – original draft. SM: Validation, Writing – review & editing. PF: Visualization, Writing – original draft. MB: Writing – review & editing. VF: Supervision, Writing – original draft.
